# Pt-grown carbon nanofibers for enzymatic glutamate biosensors and assessment of their biocompatibility†

**DOI:** 10.1039/c8ra07766e

**Published:** 2018-10-19

**Authors:** Noora Isoaho, Emilia Peltola, Sami Sainio, Jari Koskinen, Tomi Laurila

**Affiliations:** Department of Electrical Engineering and Automation, School of Electrical Engineering, Aalto University PO Box 13500 00076 Aalto Finland tomi.laurila@aalto.fi +358 50 341 4375; Department Chemistry and Materials Science, School of Chemical Technology, Aalto University PO Box 16200 00076 Aalto Finland

## Abstract

Application-specific carbon nanofibers grown from Pt-catalyst layers have been shown to be a promising material for biosensor development. Here we demonstrate immobilization of glutamate oxidase on them and their use for amperometric detection of glutamate at two different potentials. At −0.15 V *vs.* Ag/AgCl at concentrations higher than 100 μM the oxygen reduction reaction severely interferes with the enzymatic production of H_2_O_2_ and consequently affects the detection of glutamate. On the other hand, at 0.6 V *vs.* Ag/AgCl enzyme saturation starts to affect the measurement above a glutamate concentration of 100 μM. Moreover, we suggest here that glutamate itself might foul Pt surfaces to some degree, which should be taken into account when designing Pt-based sensors operating at high anodic potentials. Finally, the Pt-grown and Ni-grown carbon nanofibers were shown to be biocompatible. However, the cells on Pt-grown carbon nanofibers had different morphology and formation of filopodia compared to those on Ni-grown carbon nanofibers. The effect was expected to be caused rather by the different fiber dimensions between the samples than the catalyst metal itself. Further experiments are required to find the optimal dimensions of CNFs for biological purposes.

## Abstract 2

Pt-grown carbon nanofibers were utilized for the fabrication of glutamate biosensors and in addition their biocompatibility was assessed.

## Graphical abstract

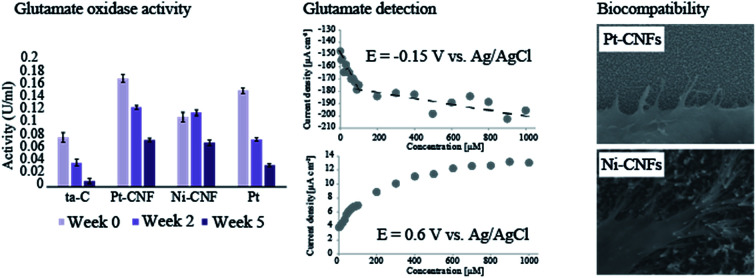

## 1. Introduction

It was stated in 2004 in the WHO Neurology Atlas^1^ that the number of cases of Parkinson's disease and other neurodegenerative conditions will probably exceed those of cancer and become the second most common cause of death. Moreover, in a research report concerning the situation in 2010 it was estimated that the costs of brain disorder in Europe including direct health care expenses, non-medical direct costs and indirect costs such as lost work time were €798 billion per year.^2^ Finally, in the second edition of the WHO Neurology Atlas it was emphasized that the burden of neurological diseases is especially high in middle- and low-income states where the accessibility to healthcare services can often be limited.^3^ In the high income countries with an aging population and longer life expectancies the costs are also likely to increase in the future. Thus, finding new means to treat the various neurological conditions is important. Moreover, increasing the knowledge of the reasons behind neurological diseases with new technologies could further help improve the lives of patients suffering from these often incurable ailments.

Etiology of neurological diseases is typically linked to neurotransmitters, such as dopamine^4^ and glutamate.^5^ These messenger molecules of the nervous system are often present in very low concentrations and can exhibit very fast transients, owing to efficient uptake mechanisms.^6,7^ Thus, in order to measure neurotransmitters the sensors have to have both high sensitivity and fast response times. Electrochemical sensors have proven to be able provide these properties.

Glutamate is the main excitatory neurotransmitter in the mammalian nervous system. It has an important role in learning and memory^8^ but on the other hand it can also have an exitotoxic effect on cells.^9^ Disrupted glutamate homeostasis has been proposed to be related to various neurological conditions, such as amyotropic lateral sclerosis (ALS), schizophrenia, epilepsy, Parkinson's disease, Alzheimer's disease, and Huntington's disease.^5,6^ Glutamate is not electrochemically active meaning that it cannot be reduced or oxidized within the water window of known electrode materials in neutral pH. Instead of direct measurements its detection is based on enzymatic sensors that utilize glutamate oxidase (GluOx) to produce electrochemically active H_2_O_2_. Thus, the requirements for glutamate sensors materials include in addition to good sensitivity to H_2_O_2_ also suitability for enzyme immobilization. Another suitable enzyme for glutamate biosensors is glutamate dehydrogenase but it requires also the immobilization of its cofactor nicotineamide dinucleotide, which makes structure more complex. In addition, non-enzymatic detection of glutamate has been shown to be possible with vertically aligned Ni nanowires.^10^ However, the reaction involves Ni (oxo)hydroxides that are stable only in alkaline pH which makes this approach unfeasible for physiological applications.

GluOx is a flavoenzyme with flavine adenine dinucleotide (FAD) as its prostetic group and it is highly specific to glutamate.^11^ The strict substrate specificity has been addressed to Arg305 residue at the active site of the enzyme.^12^ The enzymatic reaction is as follows:1
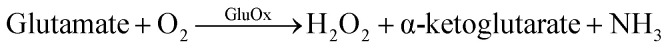


Oxygen is needed as co-substrate to re-oxidize the reduced FADH_2_ back to FAD for continuous production of H_2_O_2_.

Carbon nanofibers grown from Ni catalyst with a tetrahedral amorphous carbon (ta-C) layer functioning as an extra carbon source have been shown to be prospective candidates for detection of neurotransmitters and H_2_O_2_.^13,14^ However, as it is not possible to completely remove the Ni catalyst particles,^15^ other catalyst metals are needed in order to fabricate sensors for possible *in vivo* use where Ni could cause allergy. We have previously shown that Pt-grown carbon nanofibers (Pt-CNFs) can be utilized for H_2_O_2_ detection.^16,17^ The fibers grew as a vertically aligned forest with Pt particles located mainly at their tips. The length of the fibers varied from some tens of nanometers to hundreds of nanometers. Since the carbon nanofibers also offer suitable functional groups for covalent immobilization of enzymes we show here their application for amperometric detection of glutamate. We discuss especially the effect of detection potential and possible fouling of the Pt nanoparticles by glutamate on the results. Moreover, we present here an assessment of the materials biocompatibility based on cultures of neuronal and glial cell lines.

## 2. Materials and methods

### 2.1. Sample preparation and physical characterization

The Pt-CNF sample preparation has been described in detail in our previous publication.^16^ In brief, a 7 nm thick ta-C layer was deposited on Si with 20 nm Ti adhesion layer by utilizing dual-filtered cathodic vacuum arc deposition. Before the CNF growth a 10 nm Pt catalyst layer was deposited on top of the ta-C. Finally, CNFs were grown with plasma-enhanced chemical vapor deposition at 750 °C for 30 minutes by utilizing C_2_H_2_ as a precursor. For reference, CNFs were also grown with 20 nm Ni catalyst layer. The growth period in this case was 60 minutes. In addition, pristine 7 nm ta-C films without metal film or grown CNFs as well as 10 nm Pt thin films on Si were utilized as reference samples.

PTFE tape was utilized for defining a circular area from the samples for the electrochemical experiments.

X-ray photoelectron spectroscopy (XPS, Axis Ultra, Kratos Analytical) was utilized for estimation of carboxyl and other functional groups on the samples by peak fitting the high-resolution region for C 1s. Monochromatic Al Kα X-ray source at 100 W with charge neutralization when needed was used for collecting the spectra (0.1 e V step and 20 eV pass energy). Before measurement the samples were pre-evacuated overnight at <10^−5^ Pa. During the measurement the analysis chamber vacuum level was <10^−6^. The binding energy calibration (CO = 286.7 eV, C–C = 285.0)^18,19^ was done by utilizing cellulose filter paper, which was also used as *in situ* reference. Finally, data fitting, atomic composition ratio calculations and charge correction were performed with CasaXPS software (v 2.3.18) and utilizing equally wide half-maximum Gaussian lines for all oxygen functionalities using Shirley-background for the fit region.

### 2.2. Enzyme immobilization and activity measurements

Glutamate oxidase (GluOx, CosmoBio Co Ltd., Japan), dissolved in deionized water without further purification and stored at −80 °C, was immobilized on samples by utilizing carbodiimide crosslinking. Solution containing 0.2 M *N*-(3-(dimethylamino)propyl)-*N*′-ethylcarbodiimide (EDC, Sigma-Aldrich) and 0.05 M *N*-hydroxysuccinimide (NHS, Aldrich) was pipetted on samples and incubated at room temperature for 2 h. Reference samples without crosslinkers were prepared by pipetting only deionized water on samples. For GluOx immobilization all samples were then washed three times with phosphate buffered saline (PBS). 0.1 U ml^−1^ GluOx solution diluted in PBS was pipetted on samples and incubated for 2 h. Finally, samples were washed three times with PBS and either used in experiments or stored in PBS at +4 °C.

Enzyme activity was tested using *o*-phenylenediamine (OPD, Sigma-Aldrich) and horseradish peroxidase (HRP, Sigma-Aldrich). The product of GluOx-produced H_2_O_2_ and OPD from HRP catalyzed reaction has orange-brown color and its intensity can be read spectrophotometrically at 450 nm with a plate reader (FLUOstar Optima, BMG LABTECH). The calibration standards were prepared by diluting GluOx stock solution (10 U ml^−1^) in PBS in series (2 U ml^−1^, 0.7 U ml^−1^,…0.003 U ml^−1^) with addition of the reaction mixture containing OPD and HRP. The standards were incubated the same amount of time at the same conditions as the samples (30 min in dark at room temperature).

### 2.3. Electrochemical experiments

Electrochemical experiments were performed with a Gamry 600 or 600+ potentiostat in a three-electrode cell. The reference electrode was Ag/AgCl/KCl sat'd (Radiometer Analytical) which was separated from the measured solution by a Luggin capillary. Counter electrode was a Pt wire.

Experiments were performed in non-deaerated PBS containing NaCl (137 mM), KCl (2.7 mM), Na_2_HPO_4_ (10 mM) and KH_2_PO_4_ (1.8 mM). Fresh H_2_O_2_ and glutamate stock solutions were always prepared before experiments in PBS by using 30% H_2_O_2_ (Merck Millipore) and l-glutamic acid salt (Sigma Aldrich), respectively. Maximum solubility of l-glutamic acid in water is 8.6 mg ml^−1^ which limits the stock solution concentration.

### 2.4. Cell culture

Cell viability and morphology was examined on Pt-CNF samples and Pt thin films, Ni-CNF and ta-C were used as control samples. The samples were sterilized in 70% ethanol for 10 min prior to cell culture experiments.

Cells were cultured in humidified incubator with 5% CO_2_ in the air. C6 (ATCC® CCL-107™) rat glial cells were cultured in F12-K medium supplemented with 2.5% fetal bovine serum (FBS) and 15% horse serum. Mouse neural stem cells (mNSC, ATCC® CRL2926™) were cultured in Eagle's Minimum Essential Medium supplemented with 2 mM l-glutamine and 10% FBS. All media were supplemented with 100 IU ml^−1^ of penicillin and 100 μg ml^−1^ of streptomycin.

The seeding densities followed the recommended seeding densities for each cell type and were 64 000 cells per cm^2^ for C6 cells and 34 000 cells per cm^2^ for mNSC. The cells were cultured on samples placed on 12-well plates for 24 hours.

For actin visualization, the cells were fixed in 4% paraformaldehyde and permeabilized in 0.5% triton-X. We stained the acting cytoskeleton using phalloidin-568-label (Biotium 1 : 50 in PBS, 30 minutes incubation) and nuclei by DAPI (Vectrashield mounting medium with DAPI). We used olympus BX51M microscope and Leica DCF420 digital microscope camera for the imaging.

For scanning electron microscopy, the cells were fixed in 2.5% glutaraldehyde in PBS overnight at 4 °C. Samples were then washed with PBS and dehydrated with an increasing ethanol series and hexamethyldisilazane (Sigma-Aldrich). Completely dried samples were coated with a chromium layer to improve conductivity and inspected with a scanning electron microscope (JEOL JSM-6335F, field emission SEM).

## 3. Results and discussion

### 3.1. Enzyme immobilization and enzyme activity

**Fig. 1:**
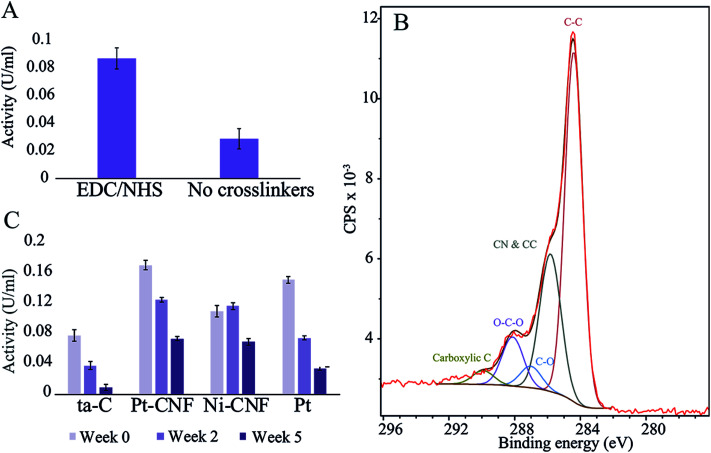
(A) GluOx activity measurements on Pt-CNFs with and without EDC/NHS crosslinking. (B) XPS spectrum and fit of the C 1s peak for Pt-CNF. (C) Enzyme activity for ta-C, Pt-CNFs, Ni-CNFs and Pt thin film at three different time points..

First, to assess the effectiveness the carbodiimide crosslinking, the enzyme activity was compared between samples where blank deionized water or 0.2 M EDC/0.05 M EDC in deionized water was utilized for the first immobilization step. Fig. 1 shows that there was a clear difference between the two sample groups with the carbodiimide crosslinkers resulting in significantly higher activity. Thus, EDC/NHS was used in all subsequent enzyme immobilizations.

The carbodiimide crosslinkers form a bond between the carboxyl groups on the sample and the amino terminals of the enzyme. We utilized XPS and fitting the C 1s peak from the spectrum in order to get an estimate of the amount of carboxyl and other functional groups on the pristine samples (Fig. 1 and Table 1). Interestingly, the amount of carboxyl groups is relatively low (1.4 ± 0.7%) despite the significant effect on the enzyme immobilization when utilizing the carbodiimide crosslinking. Specific oxygen functional groups amounts presented in Table 1 and the C 1s fit shown in Fig. 1B should be considered as approximations and used only as guidelines. Especially the non-linking carboxyl groups may be affected by the ultra-high vacuum conditions present in XPS experiments. Additionally, there were considerable variations between samples (for example the amount of carboxyl group varied from 0.5…2.5%).

**Table 1:** Estimates for the amounts of different carbon bonds in Pt-CNFs obtained from the XPS fit of C 1s. Notation: C–C: graphitic-like carbon; C–N & CC: amorphous carbon and carbon–nitrogen bonds; C–O: single bond between carbon and oxygen; C=O double bond between carbon and oxygen

C–C	C–N & CC	C–O	C=O	Carboxyl
54.7 ± 2.7%	26.8 ± 1.0%	4.2 ± 0.4%	12.7 ± 2.9%	1.4 ± 0.7%

The glutamate oxidase activity on four different substrates (plain ta-C, Pt-CNF, carbon nanofibers grown from Ni catalyst, *i.e.* Ni-CNF, and Pt thin film) was measured at three time points: immediately after immobilization (week 0), two weeks after immobilization and five weeks after immobilization. Fig. 1C shows that the Pt-CNFs showed the highest activity compared to the other samples. Interestingly, the second highest activity was obtained with Pt thin film despite it lacking the carboxyl groups required for the carbodiimide crosslinking. When the activity measurement was repeated two weeks after the immobilization, the activity for Pt-CNF had dropped by approximately 25% whereas for the Pt thin film the activity was halved. Interestingly, for the Ni-CNFs the activity stayed approximately the same. After 5 weeks the activity of Pt had decreased to about 25% of the original value. For both types of CNFs the activities were about two times higher than that on Pt and about the same order of magnitude irrespectively whether Ni or Pt fibers were considered. As there are no carboxyl groups on the Pt thin film for formation of peptide bonds through the carbodiimide crosslinking we assume that the enzyme is immobilized there *via* secondary bonds (van der Waals forces) which could explain the larger decrease in activity compared to the CNFs.

### 3.2. Glutamate detection

**Fig. 2:**
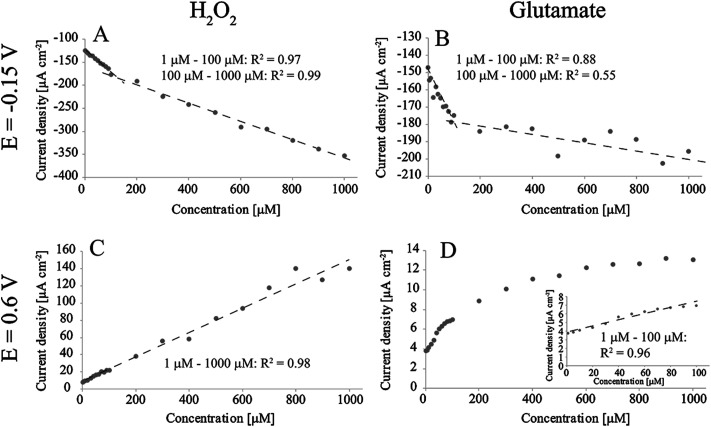
Amperometric detection of H_2_O_2_ and glutamate in non-degassed PBS with Pt-CNFs. (A) H_2_O_2_ at *E* = −0.15 V *vs.* Ag/AgCl. (B) Glutamate at *E* = −0.15 V *vs.* Ag/AgCl. (C) H_2_O_2_ at *E* = 0.6 V *vs.* Ag/AgCl. (D) Glutamate at *E* = 0.6 V *vs.* Ag/AgCl.

Fig. 2 shows the detection of H_2_O_2_ and glutamate in O_2_-containing PBS at two different potentials (−0.15 V and 0.6 V *vs.* Ag/AgCl). When measuring H_2_O_2_ at −0.15 V (Fig. 2A) there were two linear ranges (obtained by averaging current densities between 0.5 s and 1 s for each concentration): 0.464 μA μM^−1^ cm^−2^ and 0.199 μA μM^−1^ cm^−2^. We have previously discussed the reasons for the appearance of two linear ranges.^17^ Briefly, we proposed that the higher sensitivity between 1 μM and 100 μM compared to the range from 100 μM to 1000 μM arises from oxygen reduction reaction (ORR) taking place parallel to the main H_2_O_2_ reaction. ORR does not proceed to H_2_O on these samples, but instead produces additional H_2_O_2_ to the solution which in turn affects the observed current especially at low H_2_O_2_ concentrations.^17^ Similarly, there were two apparent linear ranges when measuring glutamate at the same potential: first one between 1 μM and 100 μM with sensitivity of 0.266 μA μM^−1^ cm^−2^ and the second from 100 μM to 1000 μM with sensitivity of 0.0208 μA μM^−1^ cm^−2^. However, it is debatable, when inspected more closely, whether the second range can be said to be linear. As the material has been shown to exhibit good properties for H_2_O_2_ detection, this deviation is likely to arise from the enzymatic reaction and subsequent complications related to production of hydrogen peroxide. We suggest that the observed behavior is caused by the oxygen reduction reaction consuming O_2_ from the electrode surface, which affects the enzymatic reaction (1) and accordingly hinders the production of H_2_O_2_. This becomes eminent especially at high glutamate concentrations where more oxygen would be needed to re-oxidise the FADH_2_ molecule to FAD at the GluOx active site. On the other hand, as we discussed, in addition to consuming oxygen, the ORR also produces H_2_O_2_ that can also affect the detected current. Thus, the enzyme of which function is heavily dependent on the available O_2_ is most likely the cause for lack of linearity compared to experiments where the analyte was H_2_O_2_.

For H_2_O_2_ measurement at 0.6 V there is only single linear range from 1 μM to 1000 μM: 0.142 μA μM^−1^ cm^−2^. This is in line with the previous results as there is no ORR occurring at this potential. H_2_O_2_ oxidation on Pt has been proposed to proceed by the following equations:^20^

2 2Pt(OH) + H_2_O_2_ → 2Pt(H_2_O) + O_2_

3 2Pt(H_2_O) → 2Pt(OH) + 2H^+^ + 2e^−^

Eqn (2) describes the non-electrochemical step where H_2_O_2_ is (chemically) oxidized on Pt. This step produces also O_2_ into the solution which might in turn further boost the enzyme function. The electrochemical signal arises from the reaction described by eqn (3) where the original Pt oxide sites are regenerated after being reduced during the oxidation of H_2_O_2_.

Despite the one linear range for H_2_O_2_ measurement at 0.6 V, results for glutamate detection showed linearity only up to 100 μM with sensitivity of 0.0352 μA μM^−1^ cm^−2^, which is an order of magnitude smaller compared to that for H_2_O_2_ detection. Above this concentration the current response showed saturation of GluOx. The apparent Michaelis constant (*K*_m_) for GluOx given by the supplier is 0.2 mM. If it is assumed that the concentration of the co-substrate (O_2_) remains constant during the experiment, the maximum current density response *J*_max_ can be assumed to be equal the current density at enzyme saturation.^21^ The *K*_m_ here is approximately 0.07 mM (Fig. 2D), which is considerably lower compared to that given by the supplier. When similar averaging is done later during the measurement (between 80 s and 80.5 s) the *K*_m_ value is between 0.1 mM and 0.2 mM which is more in line with the enzyme specifications. One explanation for the lower *K*_m_ in the beginning of the measurement could be that H_2_O_2_ is produced also between measurements for different concentrations while glutamate is added into solution. This additional H_2_O_2_ is then oxidized in the beginning of the experiment. When the measurement proceeds, H_2_O_2_ production becomes limited by diffusion of glutamate to the enzyme and its active site. Thus, the overall reaction kinetics becomes slower and *K*_m_ is increased. Due to the enzyme saturation and deviation from linearity it is not possible to define the glutamate sensitivity for the Pt-CNFs above 100 μM. However, it is possible to fit a logarithmic graph to the results with *R*^2^ > 0.99. On the other hand, it is often stated that the extracellular glutamate levels are in low micromolar or even submicromolar range (see for example ref. 22–24) which makes the upper limit of 100 μM sufficient.

The limit of detection (LOD) values for glutamate were defined for the Pt-CNFs as 3 × *σ*/*S*, where *σ* is the standard deviation of the signal in blank PBS and *S* is the sensitivity multiplied by the sample area. When glutamate was measured at −0.15 V *vs.* Ag/AgCl LOD was 0.3 μM whereas when the measurement was performed at 0.6 V *vs.* Ag/AgCl it was 0.7 μM (in both cases S/N = 3). There are several publications where glutamate has been measured with electrochemical sensors in different brain regions *in vivo* (including but not limited to works presented in ref. 25–34). The basal levels given vary mainly from a couple of μM to some tens of μM, even with measurements performed within the same brain region in same species. It might be that these values are, however, overestimated. Vasylieva *et al.*^32^ have stated that certain commonly utilized crosslinkers (and especially glutaraldehyde) might affect the enzyme substrate specificity resulting in too high measured concentrations. Furthermore, Herman & Jahr^24^ have proposed based on measurements in hippocampal slices that due to the fast glutamate transport systems, the basal levels could indeed be in the nanomolar range.

Considerably lower basal concentrations have been measured *in vivo* with for example liquid chromatography/tandem mass spectroscopy (rat globus pallidus 189.02 ± 14.76 nM)^35^ and high-precision liquid chromatography combined with fluorescence imaging (rat basal ganglia 107.68 ± 13.32 nM).^36^ However, the sampling in these studies was done *via* microdialysis, which suffers from low temporal resolution and the large size of the probe causing damage to the brain tissue.^37^ Moreover, it has been shown for dopamine and proposed to be applicable to other neurotransmitters as well that due to the effective uptake mechanisms the concentration of the analyte collected *in vivo* by microdialysis is lower than the actual extracellular levels.^38^ Combining the loss of neuronal activity in the vicinity of the probe and the effect of the uptake mechanisms, Kulagina *et al.*^25^ suggested that resting levels of glutamate measured *via* microdialysis are likely to be significantly underestimated, despite the low resolution of the utilized analysis methods.

Finally, because of the discrepancies in the obtained basal concentrations, it has been hypothesized that due to the differences in temporal resolution between microdialysis and electrochemical microsensors, the origin of the measured glutamate could be different and the two methods should be utilized together to complement each other.^37^

Both H_2_O_2_ and glutamate detection were also inspected at 0 V *vs.* Ag/AgCl (results not shown here). According to results presented in our earlier publication^17^ due to inhibition from chlorides there is no significant ORR occurring in PBS at 0 V. However, H_2_O_2_ is reduced at this potential. Indeed, H_2_O_2_ measurements gave only one linear range between 10 μM and 1000 μM, although with sensitivity an order of magnitude lower than that obtained at −0.15 V. However, despite the promising results obtained for H_2_O_2_ measurements, it was not possible to measure glutamate reliably at this potential as the results showed increasing current (density) instead of the expected (decreasing) response for H_2_O_2_ reduction. It is not possible to say unambiguously within the scope of this study what is behind the observed behavior. We assume that as there might be some contribution from ORR as well as possible reduction of Pt oxides. However, the overall situation is very complex and requires more thorough inspection if glutamate is to be measured at 0 V *vs.* Ag/AgCl.

**Fig. 3:**
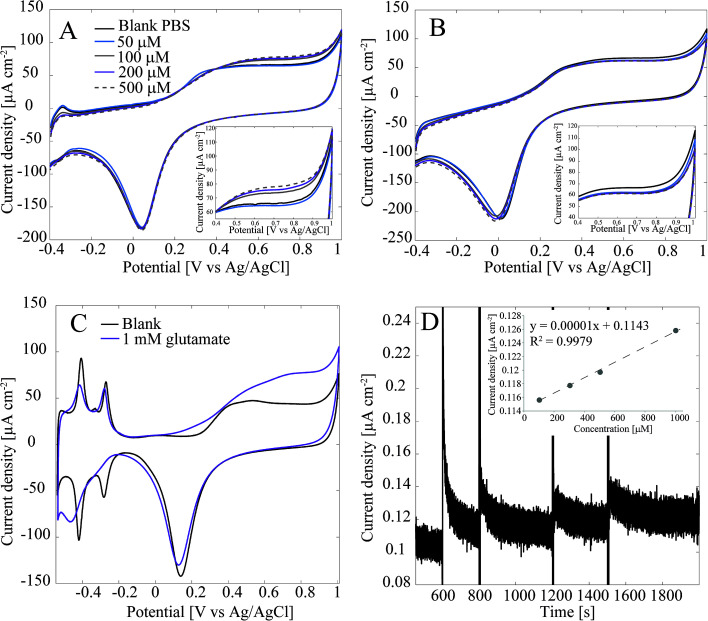
(A) Cyclic voltammogram recorded at 50 mV s^−1^ of Pt-CNF without immobilized GluOx in glutamate containing PBS (non-degassed). Inset shows the increase in current (density) in the anodic end. (B) Cyclic voltammogram recorded at 50 mV s^−1^ of Pt-CNF without immobilized GluOx in Pbs (non-degassed) with addition of blank PBS. Inset shows that there was no increase in current (density) is with addition of glutamate. (C) Flame annealed polycrystalline Pt in blank phosphate buffer (without chlorides) and with 1 mm glutamate. The originally reference electrode was RHE and the conversion to Ag/AgCl scale was done by using Nernst equation and solution pH (6.7). (D) Amperometric measurement with Pt-CNFs (no GluOx) at 0.65 V *vs.* Ag/AgCl with addition of glutamate. The inset shows that the addition of glutamate resulted in linear increase in current (density) as a function of concentration.

Detection of glutamate with the Pt-CNFs was also inspected without the enzyme. Fig. 3A shows cyclic voltammograms in blank PBS and PBS with 50 μM, 100 μM, 200 μM and 500 μM glutamate. Surprisingly, the current is increasing in the anodic end for the three largest concentrations which could indicate that glutamate is oxidized on the Pt-CNFs even without the presence of GluOx (inset in Fig. 3A). Similar cycling in PBS with additions of equal amounts of blank solution (Fig. 3B) was also carried out and corresponding increase in the current was not detected. Thus, the observed change in the current was indeed related to addition of glutamate. Electroinactive organic compounds are known to be able to adsorb on noble metals, such as Pt and Au, and their desorption from the surface during formation of metal oxides has been stated to cause anodic signals.^39^ Thus, we propose that the increase in the current does not arise from oxidation of glutamate but fouling of the Pt nanoparticles and subsequent displacement during surface oxide formation. Fig. 3C show cyclic voltammogram of flame annealed polycrystalline Pt in blank phosphate buffer (without chlorides that could be adsorbed on the surface) and phosphate buffer with 1 mM glutamate. There is significant increase in the anodic end at similar potentials compared to the Pt-CNF in glutamate solution. Moreover, the disappearance of the hydrogen adsorption peaks and diminishing desorption peaks indicate that glutamate is indeed fouling the Pt surface.^40^ Finally, amperometry at 0.65 V *vs.* Ag/AgCl was performed with the Pt-CNFs without GluOx and different amounts of glutamate stock solution (10 mM) was injected into the cell during the measurement to see if it would have an effect on the current. It is demonstrated in Fig. 3D that adding glutamate causes an increase in the current. Moreover, increase is linear in respect to increase in concentration (inset in Fig. 3D). The slope of the curve, *i.e.* sensitivity, is very low, only 0.00001 μA cm^−2^ μM^−1^ which is several orders of magnitudes lower than that obtained for H_2_O_2_ or glutamate with the immobilized enzyme. Despite the low sensitivity, this finding is significant for development of amperometric glutamate sensors based on Pt as these are often operated at anodic potentials where glutamate adsorption might contribute to the detected current. It should be noted that even though glutamate does not spontaneously form polymers, unlike for example dopamine, there are several reports where polyglutamic acid has been electropolymerized on carbon substrates.^41–46^ In all the these studies electropolymerization is facilitated by potential cycling within a relatively wide potential window (more than 2 V). It could be possible that the increase in current in Fig. 3A and C is due to polymerization of glutamate but it is not possible to discuss the mechanism further within the scope of this work.

### 3.3. Biocompatibility

When placing implants into the tissue, the typical reaction, *i.e.* host response, is the formation of fibrous capsule.^47^ In the central nervous this capsule or sheath consists of glial cells.^48^ This fibrous structure will hinder the measurement of neurotransmitters with the implanted sensors by forming a barrier between them and the electrode surface, and has been proposed as the main reason for biosensor failure *in vivo*. It has been previously shown that carbon nanomaterials can be utilized to minimize functions of glial cells.^49^ On the other hand, integration of the device into the tissue requires promoting the growth of neurons on the electrode. Reduced glial cell surface coverage while maintaining high neuronal coverage may enhance neuron-electrode coupling through nanostructure-mediated suppression of scar tissue formation.^50^ Therefore, we have here studied the growth of both glial and neural cells on the CNFs as well as on thin film samples to assess the potential of this new carbon nanomaterial in implantable devices.

**Fig. 4:**
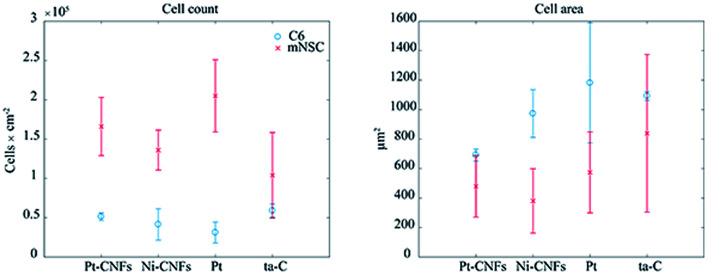
Cell count and area of glial C6 cells (blue circles) and mouse neural stem cells (red crosses) on Pt, Pt-CNFs, Ni-CNFs and ta-C.

Commonly used cytotoxicity evaluation methods, such as MTT, are not appropriate for the quantitative toxicity assessment of carbon nanostructures as MTT-formazan crystals formed in the MTT reaction are lumped with nanotubes causing false results.^51,52^ Here we evaluated cell viability by counting the cells from fluorescence microscopy images and used Ni-CNFs, Pt thin films and ta-C samples as reference samples. The number of cells is approximately the same on all the samples (Fig. 4). Based on cell count and morphology visualization, all surfaces exhibit good biocompatibility for both C6 and mNSCs.

**Fig. 5:**
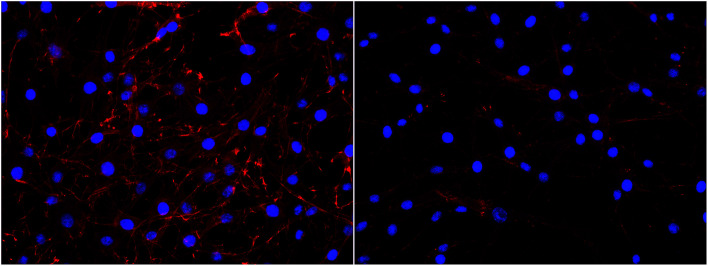
C6 cell adhesion on Pt-CNFs (left panel) and Ni-CNFs (right panel). Cell morphology is spreading on all directions on Pt-CNFs, whereas the morphology is more elongated on Ni-CNFs. The nuclei have been stained blue with DAPI and the actin cytoskeleton red with phalloidin-568-label.

**Fig. 6:**
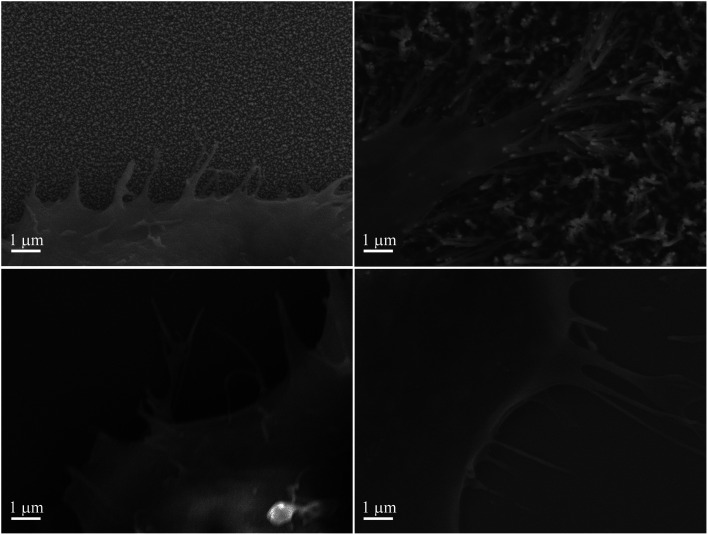
mNSC on (A) Pt-CNFs, (B) Ni-CNFs, (C) Pt thin film, and (D) ta-C. Extensive amount of filopodia is observed on Ni-CNFs compared to other tested materials.

Interestingly, a clear difference in cell adhesion and morphology can be observed between Pt-CNFs and Ni-CNFs. This effect is most likely rather due to the difference in dimension of the nanofibers than the metal involved. Pt-CNFs have a diameter range in the range from 5 nm to 45 nm, whereas those of Ni-CNFs vary from 50 to 500 nm.^53^ Further, Pt-CNFs have a height of 60–600 nm,^17^ whereas Ni-CNFs are typically taller, being closer to 1 μm. Thus, the critical dimensions of the two types of CNF structures are very different. Most evident difference originating from this feature can be seen in morphology of C6 cells (Fig. 5 and S1†). On Pt-CNFs the C6 cells are randomly spread and surface extensions protruded toward various directions, while on Ni-CNFs C6 cells possess highly elongated and oriented cell surface extensions. Both C6 (Fig. S2†) and mNSC (Fig. 6) have extensive amount of filopodias on Ni-CNFs, while their number on Pt-CNFs is only slightly increased compared to ta-C or Pt thin films.

The diameters of Ni-CNFs are similar to that of filopodia (100–300 nm^54^), and it is likely that a filopodia meets only a single Ni-CNF (or collapsed Ni-CNFs). It has been revealed that a critical lateral interdistance of about 60–70 nm between integrin arginylglycylaspartic acid (RGD) ligands exists, above which integrin clustering and focal adhesion formation is hindered, limiting cell attachment and spreading.^55^ It can be speculated that this threshold in spacing might be of physiological relevance since ordered structures occur in the native extracellular matrix, such as the 67 nm D-periodicity of collagen fibrils. As seen here, and also observed before,^56^ the CNFs tend to collapse into microbundles when dried after submersion in solution. If the fibers are collapsed already in the solution, the spacing between Ni-CNFs is larger than the above mentioned critical lateral interdistance. On the other hand, cell adhesions smaller than 1 μm^2^ appear to be able to generate large forces that do not correlate to adhesion size.^57^ This may explain the abundant number of filopodia observed on Ni-CNFs.

Pt-CNFs are smaller than Ni-CNFs and consequently cell filopodia meets multiple Pt-CNF fibers and is more flexible to move. This nm-scale stimulus may also promote the formation of proper synaptic connections. Indeed, it has been observed that astrocytic syncytium level maximized at 50 nm tantalum nitride dot arrays, when dot diameters range from 10 to 200 nm were compared.^58^ In the point of view of cell response, thin fibers (a few dozen nm) may provide better response than thick >100 nm fibers. However, even though the C6 cells on Pt-CNFs seem to be well spread (Fig. 5 and S1†), their size is smaller than C6 on Ni-CNFs, ta-C or Pt thin films (Fig. 4). As the size of CNFs clearly alters the cell morphology and size, it is clear that further effects on cell metabolisms and functions will occur. Further examination on these issues are required. The biocompatibility of vertically aligned CNFs has not been widely assessed. We have shown that CNFs grown from ta-C without any additional coating layers providing support for the biocompatibility for C6 rat glial cells^14^ and other others have shown that PC12 neuron cells cultured on CNF structures can form extended neural network upon proper chemical and biochemical modifications^56^ and that CNFs can be used for the entrapment of colon cancer cells with different metastatic grades.^59–61^ Many parameters, such as length, diameter and separation of nanofibers as well as the structure (*e.g.* bamboo, stacked or fishbone) and the utilized catalyst may influence cellular response.

Also, the growth of CNFs is a stochastic process, resulting in highly heterogeneous structure, which further complicates the understanding of the interaction of cells and CNFs. However, this heterogeneous structure may provide more natural signals for cells than highly ordered nanostructures.

From electrochemical point of view, long fibers increase electrochemically active area. To optimize the structure, the fibers should be high, but the aspect ratio such that the fibers do not collapse due to large capillary forces^62^ and the fibers retain their original free-standing structure. From biological point of view, both tested CNFs exhibited good biocompatibility, but further examination is required to understand the effects of different fiber sizes on cells to find the optimal dimensions for biological response. An interesting aspect to study in the future is to find means to promote the growth of neural cells and prevent that of glial cells. The formation of glial scar or encapsulation around the enzyme isolates the sensor from the surroundings preventing analytes from reaching the its surface.^63,64^ Neurons, on the other hand, should grow as near the sensor surface as possible to minimize the diffusion distance for the target neurotransmitters. We have already shown that the surface chemistry of carbon nanodiamonds affects the type of cells grown on samples.^65^ Our hypothesis is that similar results could be achieved by tailoring the CNFs structure and morphology.

## 4. Conclusions

We have presented here the results describing the immobilization of GluOx on Pt-grown carbon nanofibers and electrochemical detection of glutamate with them. The Pt-CNFs showed enhanced enzyme activity after 5 weeks from immobilization when compared to carbon and Pt thin films. When measuring glutamate amperometrically at −0.15 V *vs.* Ag/AgCl there were two linear ranges: first one between 1 μM and 100 μM with sensitivity of 0.266 μA μM^−1^ cm^−2^ and the second between 100 μM to 1000 μM with sensitivity of 0.0208 μA μM^−1^ cm^−2^, which was in line with results from H_2_O_2_ experiments with the Pt-CNFs. However, the linearity for the higher range was significantly affected by oxygen reduction reaction consuming O_2_ from the vicinity of the sensor surface and consequently interfering with the production of H_2_O_2_. When the measurement was performed at 0.6 V *vs.* Ag/AgCl similar effect was not observed. Instead, there results show enzyme saturation above 100 μM and it was not possible to define linear range for higher concentrations. It can be concluded, that the Pt-CNFs are suitable for glutamate detection at −0.15 and 0.6 V *vs.* Ag/AgCl at concentrations below 100 μM. In addition we inspected the effect of possible glutamate adsorption and showed that it might affect the response obtained with Pt-based sensors especially at anodic potentials.

Finally, we studied the biocompatibility of the Pt-CNFs *via* cell culture experiments with C6 and mNSC cell lines. According to cell count and morphology visualization the samples showed good biocompatibility. When compared to CNFs grown from Ni catalyst the Pt-CNFs showed significantly different morphology and formation of filopodia for C6 cell line. This observation was suggested to arise rather from the different dimensions between the two types of CNFs than the different catalyst metals. To understand the effect of the fiber size and to obtain optimal dimensions for biological response, further studies should be conducted in the future.

## Conflicts of interest

There are no conflicts of interest to declare.

## Supplementary Material

RA-008-C8RA07766E-s001

## References

[cit1] World Health Organization , Atlas: Country Resources for Neurological Disorders, 2004

[cit2] Gustavsson A., Svensson M., Jacobi F., Allgulander C., Alonso J., Beghi E., Dodel R., Ekman M., Faravelli C., Fratiglioni L., Gannon B., Jones D. H., Jennum P., Jordanova A., Jönsson L., Karampampa K., Knapp M., Kobelt G., Kurth T., Lieb R., Linde M., Ljungcrantz C., Maercker A., Melin B., Moscarelli M., Musayev A., Norwood F., Preisig M., Pugliatti M., Rehm J., Salvador-Carulla L., Schlehofer B., Simon R., Steinhausen H.-C., Stovner L. J., Vallat J.-M., Van den Bergh P., van Os J., Vos P., Xu W., Wittchen H.-U., Jönsson B., Olesen J. (2011). Cost of disorders of the brain in Europe 2010. Eur. Neuropsychopharmacol..

[cit3] World Health Organization , Atlas: Country Resources for Neurological Disorders, 2nd edn, 2017

[cit4] Beaulieu J.-M., Gainetdinov R. R. (2011). The physiology, signaling, and pharmacology of dopamine receptors. Pharmacol. Rev..

[cit5] Meldrum B. S. (2000). Glutamate as a neurotransmitter in the brain: review of physiology and pathology. J. Nutr..

[cit6] Danbolt N. C. (2001). Glutamate uptake. Prog. Neurobiol..

[cit7] Robinson D. L., Hermans A., Seipel A. T., Wightman R. M. (2008). Monitoring rapid chemical communication in the brain. Chem. Rev..

[cit8] Riedel G. (2003). Glutamate receptor function in learning and memory. Behav. Brain Res..

[cit9] Zhou Y., Danbolt N. C. (2014). Glutamate as a neurotransmitter in the healthy brain. J. Neural Transm..

[cit10] Jamal M., Hasan M., Mathewson A., Razeeb K. M. (2013). Disposable sensor based on enzyme-free Ni nanowire array electrode to detect glutamate. Biosens. Bioelectron..

[cit11] Kusakabe H., Midorikawa Y., Fujishima T., Kuninaka A., Yoshino H. (1983). Purification and properties of a new enzyme, L-glutamate oxidase, from *Streptomyces* sp. X-119-6 grown on wheat bran. Agric. Biol. Chem..

[cit12] Utsumi T., Arima J., Sakaguchi C., Tamura T., Sasaki C., Kusakabe H., Sugio S., Inagaki K. (2012). Arg305 of *Streptomyces* L-glutamate oxidase plays a crucial role for substrate recognition. Biochem. Biophys. Res. Commun..

[cit13] Sainio S., Palomäki T., Tujunen N., Protopopova V., Koehne J., Kordas K., Koskinen J., Meyyappan M., Laurila T. (2015). Integrated carbon nanostructures for detection of neurotransmitters. Mol. Neurobiol..

[cit14] Isoaho N., Peltola E., Sainio S., Wester N., Protopopova V., Wilson B. P., Koskinen J., Laurila T. (2017). Carbon nanostructure based platform for enzymatic glutamate biosensors. J. Phys. Chem. C.

[cit15] Sainio S., Nordlund D., Gandhiraman R., Jiang H., Koehne J., Koskinen J., Meyyappan M., Laurila T. (2016). What nitric acid really does to carbon nanofibers?. J. Phys. Chem. C.

[cit16] Laurila T., Sainio S., Jiang H., Isoaho N., Koehne J. E., Etula J., Koskinen J., Meyyappan M. (2017). Application-specific catalyst layers: Pt-containing carbon nanofibers for hydrogen peroxide detection. ACS Omega.

[cit17] Isoaho N., Sainio S., Wester N., Botello L., Johansson L.-S., Peltola E., Climent V., Feliu J. M., Koskinen J., Laurila T. (2018). Pt-grown carbon nanofibers for detection of hydrogen peroxide. RSC Adv..

[cit18] Beamson G. and Briggs D., High resolution XPS of organic polymers, Wiley, Chichester, 1992

[cit19] Johansson L.-S., Campbell J. M. (2004). Reproducible XPS on biopolymers: cellulose studies. Surf. Interface Anal..

[cit20] Katsounaros I., Schneider W. B., Meier J. C., Benedikt U., Biedermann P. U., Auer A. A., Mayrhofer K. J. J. (2012). Hydrogen peroxide electrochemistry on platinum: Towards understanding the oxygen reduction reaction mechanism. Phys. Chem. Chem. Phys..

[cit21] McMahon C. P., Rocchitta G., a Serra P., Kirwan S. M., Lowry J. P., O'Neill R. D. (2006). The efficiency of immobilised glutamate oxidase decreases with surface enzyme loading: An electrostatic effect, and reversal by a polycation significantly enhances biosensor sensitivity. Analyst.

[cit22] Herrera-Marschitz M., You Z.-B., Goiny M., Meana J. J., Silveira R., V Godukhin O., Chen Y., Espinoza S., Pettersson E., Loidl C. F., Lubec G., Andersson K., Nylander I., Terenius L., Ungerstedt U. (1996). On the origin of extracellular glutamate levels monitored in the basal ganglia of the rat by *in vivo* microdialysis. J. Neurochem..

[cit23] Attwell D. (2000). Brain uptake of glutamate: food for thought 1,2. J. Nutr..

[cit24] Herman M. A., Jahr C. E. (2007). Extracellular glutamate concentration in hippocampal slice. J. Neurosci..

[cit25] Kulagina N. V., Shankar L., Michael A. C. (1999). Monitoring glutamate and ascorbate in the extracellular space of brain tissue with electrochemical microsensors. Anal. Chem..

[cit26] Oldenziel W. H., Dijkstra G., Cremers T. I. F. H., Westerink B. H. C. (2006). *In vivo* monitoring of extracellular glutamate in the brain with a microsensor. Brain Res..

[cit27] Qin S., Van Der Zeyden M., Oldenziel W. H., Cremers T. I. F. H., Westerink B. H. C. (2008). Microsensors for *in vivo* measurement of glutamate in brain tissue. Sensors.

[cit28] Day B. K., Pomerleau F., Burmeister J. J., Huettl P., Gerhardt G. A. (2006). Microelectrode array studies of basal and potassium-evoked release of L-glutamate in the anesthetized rat brain. J. Neurochem..

[cit29] Rutherford E. C., Pomerleau F., Huettl P., Strömberg I., Gerhardt G. A. (2007). Chronic second-by-second measures of L-glutamate in the central nervous system of freely moving rats. J. Neurochem..

[cit30] Mattinson C. E., Burmeister J. J., Quintero J. E., Pomerleau F., Huettl P., Gerhardt G. A. (2011). Tonic and phasic release of glutamate and acetylcholine neurotransmission in sub-regions of the rat prefrontal cortex using enzyme-based microelectrode arrays. J. Neurosci. Methods.

[cit31] Stephens M. L., Quintero J. E., Pomerleau F., Huettl P., Gerhardt G. A. (2011). Age-related changes in glutamate release in the CA3 and dentate gyrus of the rat hippocampus. Neurobiol. Aging.

[cit32] Vasylieva N., Maucler C., Meiller A., Viscogliosi H., Lieutaud T., Barbier D., Marinesco S. (2013). Immobilization method to preserve enzyme specificity in biosensors: consequences for brain glutamate detection. Anal. Chem..

[cit33] Hascup K. N., Hascup E. R., Pomerleau F., Huettl P., a Gerhardt G. (2008). Second-by-second measures of L-glutamate in the prefrontal cortex and striatum of freely moving mice. J. Pharmacol. Exp. Ther..

[cit34] Quintero J. E., Day B. K., Zhang Z., Grondin R., Stephens M. L., Huettl P., Pomerleau F., Gash D. M., Gerhardt G. A. (2007). Amperometric measures of age-related changes in glutamate regulation in the cortex of rhesus monkeys. Exp. Neurol..

[cit35] Buck K., Voehringer P., Ferger B. (2009). Rapid analysis of GABA and glutamate in microdialysis samples using high performance liquid chromatography and tandem mass spectrometry. J. Neurosci. Methods.

[cit36] Ochi M., Shiozaki S., Kase H. (2004). Adenosine A2A receptor-mediated modulation of GABA and glutamate release in the output regions of the basal ganglia in a rodent model of Parkinson's disease. Neuroscience.

[cit37] van der Zeyden M., Oldenziel W. H., Rea K., Cremers T. I., Westerink B. H. (2008). Microdialysis of GABA and glutamate: Analysis, interpretation and comparison with microsensors. Pharmacol., Biochem. Behav..

[cit38] Yang H., Peters J. L., Michael A. C. (1998). Coupled effects of mass transfer and uptake kinetics on *in vivo* microdialysis of dopamine. J. Neurochem..

[cit39] Johnson D. C., LaCourse W. R. (1990). Liquid Chromatography with Pulsed Electrochemical Detection at Gold and Platinum Electrodes. Anal. Chem..

[cit40] Climent V., Feliu J. M. (2011). Thirty years of platinum single crystal electrochemistry. J. Solid State Electrochem..

[cit41] Yu A.-M., Chen H.-Y. (1997). Electrocatalytic oxidation and determination of ascorbic acid at poly(glutamic acid) chemically modified electrode. Anal. Chim. Acta.

[cit42] Santos D. P., Bergamini M. F., Fogg A. G., Zanoni M. V. B. (2005). Application of a glassy carbon electrode modified with poly(glutamic acid) in caffeic acid determination. Microchim. Acta.

[cit43] Santos D. P., Zanoni M. V. B., Bergamini M. F., Chiorcea-Paquim A. M., Diculescu V. C., Oliveira Brett A. M. (2008). Poly(glutamic acid) nanofibre modified glassy carbon electrode: characterization by atomic force microscopy, voltammetry and electrochemical impedance. Electrochim. Acta.

[cit44] Zhang Y., Luo L., Ding Y., Liu X., Qian Z. (2010). A highly sensitive method for determination of paracetamol by adsorptive stripping voltammetry using a carbon paste electrode modified with nanogold and glutamic acid. Microchim. Acta.

[cit45] Liu X., Luo L., Ding Y., Ye D. (2011). Poly-glutamic acid modified carbon nanotube-doped carbon paste electrode for sensitive detection of L-tryptophan. Bioelectrochemistry.

[cit46] Ganesh P. S., Swamy B. E. K. (2015). Simultaneous electroanalysis of norepinephrine, ascorbic acid and uric acid using poly(glutamic acid) modified carbon paste electrode. J. Electroanal. Chem..

[cit47] Wisniewski N., Reichert M. (2000). Methods for reducing biosensor membrane biofouling. Colloids Surf., B.

[cit48] Turner J. N., Shain W., Szarowski D. H., Andersen M., Martins S., Isaacson M., Craighead H. (1999). Cerebral astrocyte response to micromachined silicon implants. Exp. Neurol..

[cit49] McKenzie J. L., Waid M. C., Shi R., Webster T. J. (2004). Decreased functions of astrocytes on carbon nanofiber materials. Biomaterials.

[cit50] Chapman C. A. R., Chen H., Stamou M., Biener J., Biener M. M., Lein P. J., Seker E. (2015). Nanoporous gold as a neural interface coating: effects of topography, surface chemistry, and feature size. ACS Appl. Mater. Interfaces.

[cit51] Casey A., Herzog E., Davoren M., Lyng F. M., Byrne H. J., Chambers G. (2007). Spectroscopic analysis confirms the interactions between single walled carbon nanotubes and various dyes commonly used to assess cytotoxicity. Carbon N. Y..

[cit52] Wörle-Knirsch J. M., Pulskamp K., Krug H. F. (2006). Oops they did it again! Carbon nanotubes hoax scientists in viability assays. Nano Lett..

[cit53] Sainio S., Jiang H., Caro M. A., Koehne J., Lopez-Acevedo O., Koskinen J., Meyyappan M., Laurila T. (2016). Structural morphology of carbon nanofibers grown on different substrates. Carbon N. Y..

[cit54] Mattila P. K., Lappalainen P. (2008). Filopodia: molecular architecture and cellular functions. Nat. Rev. Mol. Cell Biol..

[cit55] Arnold M., Cavalcanti-Adam E. A., Glass R., Blümmel J., Eck W., Kantlehner M., Kessler H., Spatz J. P. (2004). Activation of integrin function by nanopatterned adhesive interfaces. ChemPhysChem.

[cit56] Nguyen-Vu T. D. B., Chen H., Cassell A. M., Andrews R. J., Meyyappan M., Li J. (2007). Vertically aligned carbon nanofiber architecture as a multifunctional 3-D neural electrical interface. IEEE Trans. Biomed. Eng..

[cit57] Tan J. L., Tien J., Pirone D. M., Gray D. S., Bhadriraju K., Chen C. S. (2003). Cells lying on a bed of microneedles: an approach to isolate mechanical force. Proc. Natl. Acad. Sci. U. S. A..

[cit58] Lee C.-H., Cheng Y.-W., Huang G. (2014). Topographical control of cell-cell interaction in C6 glioma by nanodot arrays. Nanoscale Res. Lett..

[cit59] Abdolahad M., Sanaee Z., Janmaleki M., Mohajerzadeh S., Abdollahi M., Mehran M. (2012). Vertically aligned multiwall-carbon nanotubes to preferentially entrap highly metastatic cancerous cells. Carbon N. Y..

[cit60] Abdolahad M., Taghinejad M., Taghinejad H., Janmaleki M., Mohajerzadeh S. (2012). A vertically aligned carbon nanotube-based impedance sensing biosensor for rapid and high sensitive detection of cancer cells. Lab Chip.

[cit61] Abdolahad M., Mohajerzadeh S., Janmaleki M., Taghinejad H., Taghinejad M. (2013). Evaluation of the shear force of single cancer cells by vertically aligned carbon nanotubes suitable for metastasis diagnosis. Integr. Biol..

[cit62] Bico J., Roman B., Moulin L., Boudaoud A. (2004). Adhesion: elastocapillary coalescence in wet hair. Nature.

[cit63] Polikov V. S., Tresco P. A., Reichert W. M. (2005). Response of brain tissue to chronically implanted neural electrodes. J. Neurosci. Methods.

[cit64] Salatino J. W., Ludwig K. A., Kozai T. D. Y., Purcell E. K. (2017). Publisher correction: glial responses to implanted electrodes in the brain. Nat. Biomed. Eng..

[cit65] Peltola E., Wester N., Holt K. B., Johansson L., Koskinen J., Myllymäki V., Laurila T. (2017). Nanodiamonds on tetrahedral amorphous carbon significantly
enhance dopamine detection and cell viability. Biosens. Bioelectron..

