# Supplementary material for: Pt-grown carbon nanofibers for enzymatic glutamate biosensors and assessment of their biocompatibility
Source: RSC Adv. 2018 Oct 19;8(62):35802–12. doi: 10.1039/c8ra07766e (PMC9088215; doi:10.1039/c8ra07766e)
Supplement: RA-008-C8RA07766E-s001 [file RA-008-C8RA07766E-s001.pdf]

Electronic Supplementary Material (ESI) for RSC Advances.
This journal is © The Royal Society of Chemistry 2018

Supplementary information

Pt-grown carbon nanofibers for enzymatic glutamate biosensors and assessment of their biocompatibility

Noora Isoaho[a], Emilia Peltola[a], Sami Sainio[b], Jari Koskinen[b], Tomi Laurila[a*]

[a] Department of Electrical Engineering and Automation, School of Electrical Engineering, Aalto University, PO Box 13500, 00076 Aalto, Finland

[b] Department Chemistry and Materials Science, School of Chemical Technology, Aalto University, PO Box 16200, 00076 Aalto, Finland

*Corresponding author: tomi.laurila@aalto.fi, +358 50 341 4375

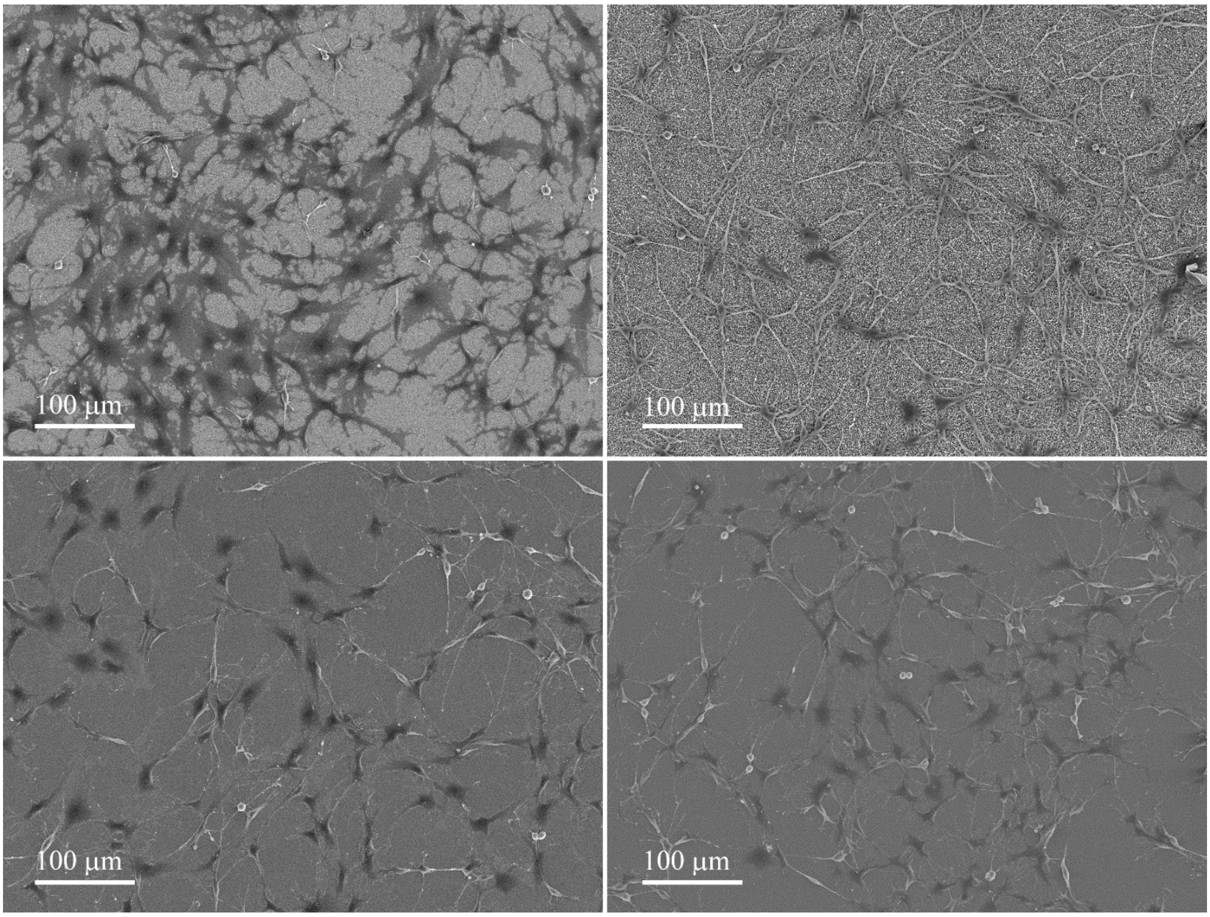

Supplementary image 1: C6 cells on a) Pt-CNFs, b) Ni-CNFs, c) Pt thin film, and d) ta-C. Cell morphology is spreading on all directions on Pt-CNFs, whereas on other tested materials the morphology is more elongated.

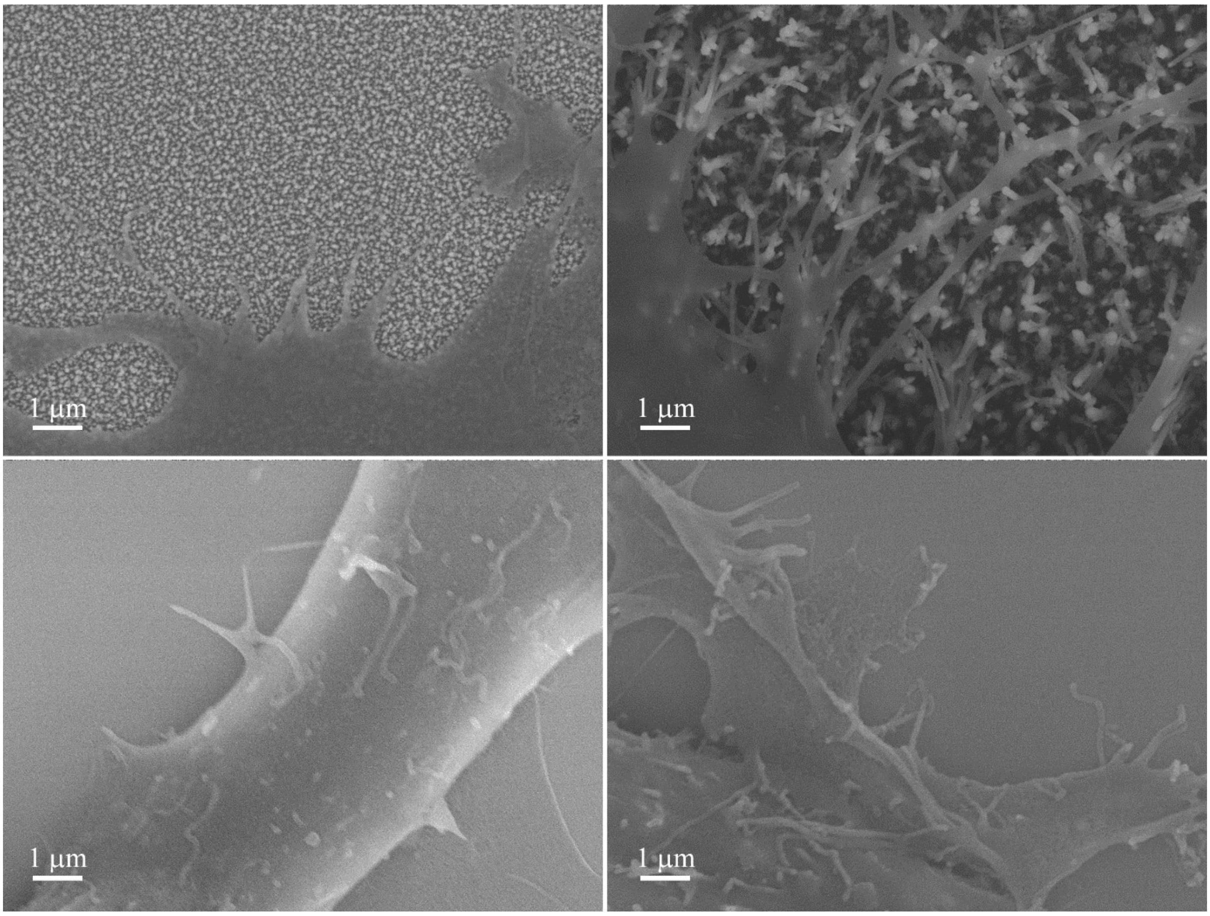

Supplementary image 2: C6 cells on a) Pt-CNF, b) Ni-CNF, c) Pt thin film, and d) ta-C. Extensive amount of filopodia is observed on Ni-CNFs compared to other tested materials.
